# Suicidal behavior and associated factors among holy water users at Northwest, Ethiopia, 2023: an institution based cross-sectional study

**DOI:** 10.3389/fpsyt.2024.1398363

**Published:** 2024-05-28

**Authors:** Gedefaw Tegegne Kassahun, Fikir Addis, Tilahun Belete Mossie, Habte Belete, Birhanu Mengist Munie

**Affiliations:** ^1^ Department of Psychiatry, Debre Markos Comprehensive Specialized Hospital, Amhara Regional Health Bureau, Debre Markos, Ethiopia; ^2^ Department of Psychiatry, College of Medicine and Health Sciences, Bahir Dar University, Bahir Dar, Ethiopia; ^3^ Department of Psychiatry, College of Medicine and Health Sciences, Debre Tabor University, Debre Tabor, Ethiopia

**Keywords:** depression, ethiopia, holy water, suicidal behavior, suicide

## Abstract

**Background:**

Suicide is a serious cause of mortality that affects families, communities, and the entire country. Because of a lack of national systematic reporting for cause-specific mortality, a high level of stigma, and religious non-acceptance, suicidal behavior is an under-reported and concealed cause of death in the majority of low- and middle-income countries.

**Objective:**

The aim of this study was to assess the prevalence of suicidal behavior and associated factors among holy water users at the Andassa Saint George Monastery, 2023.

**Methods:**

An institution-based cross-sectional study was conducted at the Andassa Saint George Monastery from 5 April to 5 May 2023. A systematic random sampling method was utilized to select 423 study participants and the Suicidal Behaviors Questionnaire—Revised was used to assess suicidal behavior. The data were gathered using the epicollect5 software with a face-to-face interview method then exported to SPSS-25 for analysis. A binary logistic regression model was used and all variables in a bivariate analysis with a *p*-value of less than 0.25 were entered into a multivariable logistic regression model, and statistical significance was declared at a *p*-value of less than 0.05.

**Results:**

The prevalence of suicidal behavior among holy water users was 9.7% (95% CI: 7.1–12.4). Being female [2.632 (1.206–5.748)], living alone [2.52 (1.06–5.97)], and having depression [3.03 (1.32–6.99)], epilepsy [3.82 (1.28–11.40)], and diabetes mellitus [3.37 (1.229–9.25)] were significantly associated with suicidal behavior.

**Conclusion:**

In this study, almost 1 in 10 had engaged in suicidal behavior in their lifetime. Several risk factors for suicidal behavior were identified, including being female, living alone, and having diabetes mellitus, epilepsy, and depression.

## Background

According to the World Health Organization (WHO), suicide is an intentional act of self-harm carried out by a person who is fully aware of or anticipates death. It is a condition in which options or choices are never considered prior to the action, it involves the purposeful or voluntary decision to end one’s life, the person’s readiness to die comes from inside him, and it is brought on by the existence of a known or hidden reason (1).

The *Diagnostic and Statistical Manual of Mental Disorders, Fifth Edition* (*DSM-5*) defines suicidal ideation as thoughts about self-harm with deliberate consideration or planning of possible techniques of causing one’s own death, while suicide is the act of intentionally causing one’s own death, and a suicide attempt is an attempt to end one’s own life, which may lead to one’s death (2).

The concept of a “suicidal process” classifies suicidal behavior chronologically; this process starts with suicidal ideation and then implies a progression of suicidality ranging from suicidal ideation over plans to suicide attempts and finally fatal suicide (3).

Suicidal behavior includes the tendency, thoughts, or acts of self-harming behavior or life-threatening risks. Suicidal behavior can be either direct (such as suicidal ideation, suicide attempt, or completed suicide) or indirect (such as risky driving, high-risk hobbies, hazardous alcohol drinking, drug misuse, or neglecting the management of physical illness) (4).

The development of suicide risk is complex, involving contributions from biological (including genetics), psychological (such as certain personality traits), clinical (such as comorbid psychiatric illness), social, and environmental factors. The involvement of multiple risk factors in conveying the risk of suicide means that determining an individual’s risk of suicide is challenging (5).

Suicidal ideation, planning, and attempt are among the three most commonly studied non-fatal suicidal behaviors that vary on a continuum of severity. While suicidal ideation is more common, this milder form of suicidal behavior is often thought to occur prior to suicide planning and attempt (6). According to available evidence, suicidal ideation can be regarded as a trigger for attempted and completed suicide (7).

A suicide plan is defined as a proposed method of carrying out a design that will result in a potentially self-injurious outcome, or a systematic formulation of a program of action that may result in self-injury (8). It is an important phase in the suicidal process preceding attempted suicide, which is one of the major risk factors for completed suicide (9).

Suicidal attempt is an intentional but unsuccessful act of killing oneself, and for every person who dies by suicide, many more attempt it (10).

The development of suicide risk is complex, involving contributions from biological (including genetics), psychological (such as certain personality traits), clinical (such as comorbid psychiatric illness), social, and environmental factors. The involvement of multiple risk factors in conveying the risk of suicide means that determining an individual’s risk of suicide is challenging (5).

The WHO mortality database indicates over 800,000 annual suicide deaths worldwide, making it one of the leading causes of death (11). It is the fourth leading cause of death among people 35–44 years of age, the fifth leading cause among people ages 45–54, and the eighth leading cause among people 55–64 years of age (12).

People with mental disorders and psychological distress have been found to be more likely to engage in suicidal behavior, and experiencing adverse life events, unemployment, lower socioeconomic status, female sex, younger age, and the suicide of a close relative have been associated with suicidal behavior (13).

Many individuals who have suicide ideation/attempts have clinical depression or subclinical symptoms of depression and showed high intent for suicide (14).

Suicidal thoughts and plans (suicidal ideation) and suicide attempts are frequently the prodromal for later suicide and indicate significant personal distress and psychological burden. It is crucial to understand the patterns of suicidal ideation and suicide attempts before implementing effective suicide prevention measures. In developed countries, the lifetime prevalence of suicidal ideation and attempt is approximately 3.0%–15.9% and 0.5%–5%, respectively, with women at higher risk than men (15).

According to the WHO, a person commits suicide every 40 s somewhere in the world, and a person attempts to die every 3 s, and according to self-report, for every suicide death, 20 people attempt suicide (5).

Suicide ideation, which comprises suicidal thoughts or threats devoid of action, is more common than suicide attempts and completed suicides, and its prevalence varies widely. The lifetime prevalence of suicidal ideation has been reported to range from 2% to 18% (16).

Culture is a dynamic collection of customs, traditions, and values to which a community or society ascribes, which may strongly influence an individual’s perception of suicide (17). Specifically, cultural values and societal structures affect how a person perceives circumstances as risk and protective factors. For example, in some studies, religiosity has been shown to be a protective factor for suicidality, through increased social integrations, as well as hope created by religious beliefs, especially in areas with high religious homogeneity (18). Some cultures completely censure suicide and view it as an abominable act; others may have some level of permissiveness, while others may view it as an honorable act. Moreover, the meaning and consequence of a suicidal act are heavily influenced by cultural norms of a society (19, 20).

Suicide motivation and permissiveness differed by age group, with the older age group likely to report loneliness, abandonment, and existing chronic illnesses as precipitants of suicide, while the younger age group was reported to be triggered by interpersonal problems and financial strain as potential stressors contributing to suicidality (21).

According to surveys carried out in 17 countries, the risk of each suicidal behavior is significantly related to being female, being younger in age, having fewer years of formal education, and never having been married (22).

Most suicides are related to psychiatric disease, with depression, substance use disorders, and psychosis being the most relevant risk factors, and most people who have died by suicide have suffered from a mental disorder; surprisingly, most of the patients with mental illness in Ethiopia visit healthcare facilities for traditional treatments (23, 24).

According to the study, suicide is also common in chronic medial illness; the magnitude of suicidal behavior among patients with diabetes mellitus (DM) at Northwest Ethiopia was 30.8%, 15.8% had suicidal ideation, 14.4% had a suicidal attempt, and 15.1% of them had a plan to commit suicide (25).

Studies also suggest a variety of risk factors for suicidal ideation, such as previous suicide attempts (26). Low social support, substance use disorder, hopelessness, sleep disturbances (27), and elevated inflammation have been found to be associated with suicidal ideation (28).

According to estimates, 75% of suicides occur in low- and middle-income countries, where limited resources are available to prevent suicidal behavior. According to data on non-fatal suicidal behaviors, including ideation, suicidal behaviors are also a leading cause of mortality and injury that affect a wide variety of age groups in several countries (29).

The global suicide rate has increased by 60% over the last 45 years, with the population aged 15–34 being the most vulnerable to becoming victims of suicide. It is a huge but largely preventable health problem, causing almost half of all violent deaths and resulting in one million fatalities each year, as well as economic costs in the billions of dollars (30).

The magnitude of suicidal behavior was very high among people with mental illness like major depressive disorders and other comorbid psychiatric disorders, and having comorbid substance use disorders was a predictor of suicidal behaviors (31).

Globally, 50% of all violent deaths in men and 71% of deaths in women were accounted for by suicide, and rates are highest among individuals aged 70 and older for both men and women in almost all regions of the world (32).

Suicide is a sensitive but under-recognized fatal health problem in Ethiopia. In addition to religious beliefs, the majority of people in Ethiopia use holy water (spiritual practice) for various mental and physical health problems. The majority of patients suffer from serious mental illnesses, such as mood disorders (30%), substance abuse (24%), and schizophrenia (40%), and the majority of patients (92.2%) were comforted by the combination of medication, holy water treatment, and prayers, and 73.6% thought that their disease was the result of an evil spirit possessing them, which is why many suicidal cases use holy water instead of getting modern treatment (33).

Public and private religious practices not only help maintain mental health and prevent mental health diseases but also help individuals cope up with their anxiety, fears, frustration, anger, anomie, feelings of inferiority, despondency, and isolation; thus, there is a high burden of suicide and mental illness in the use of holy water (34).

In Ethiopia, for example, less than 10% of people with severe mental illness had contact with modern psychiatric services and 15% to 20% of people who attend general medical clinics do so because of mental disorders, although their mental health problems are often not recognized (35, 36).

According to studies, more Ethiopians prefer the services of informal caregivers, such as family, friends, and traditional healers, over those of biomedical health centers when it comes to managing severe medical conditions, especially mental health including suicide (37).

In the Ethiopian Orthodox Church (EOTC), the primary form of well-accepted traditional religious–magical healing practice is the holy water (“Tsebel”); one may drink blessed holy water to heal any form of illness including suicide and physical diseases. As a result, assessing suicidal behavior and its associated factors among holy water users is critical to understand its magnitude, develop intervention strategies, and minimize or prevent suicidal behavior among holy water users.

It is also important for the EOTC to be informed about the practices in relation to mental healthcare including suicidal behavior so that they can make informed decisions about their choice in mental health service providers.

The burden of suicide constitutes a serious public health issue worldwide that needs the help of mental health professionals to increase awareness towards suicide warning signs. Suicide warning signs are associated with acute factors that inform clinicians about observable signs and expressed emotions and are important for saving lives through early detection and intervention for those at risk.

## Materials and methods

### Study area and period

This study was conducted from 5 April to 5 May 2023 among holy water users at the Andassa Saint George Monastery. The Andassa Saint George Monastery is an Orthodox Church established in 1640 and found in the Amhara region of northwestern Ethiopia, 20 km from Bahir Dar. Approximately 20,280 people per year come to the monastery from different places to use holy water for different physical and mental illnesses as well as for religious purposes (38).

### Study design

An institution-based cross-sectional study design was employed.

### Population

#### Source population

All adults using holy water at the Andassa Saint George Monastery were the source population.

#### Study population

All adults using holy water at the Andassa Saint George Monastery during the study period comprise the study population.

#### Sample population

Adults using holy water at the Andassa Saint George Monastery who fulfill the inclusion criteria were the sample population.

### Eligibility criteria

#### Inclusion criteria

All individuals 18 years and older who use holy water at the Andassa George Monastery in the Amhara region who gave informed consent were eligible to participate in the study.

#### Exclusion criteria

People who were unable to communicate and people with hearing disability were not included in the study. People who were severely ill during the data collection period were also not included in the study.

### Sample size determination and sampling technique

#### Sample size determination

The sample size was calculated by using the single population proportion formula: *n* =*z*2*pq*/*d*2, considering the following assumptions: prevalence *p* = 50% because no similar study was performed among holy water users in Ethiopia, with 95% confidence interval, a margin of error 5%, and a non-response rate of 10%.

*n* = sample size, *z* =standard normal distribution, and *p* = estimated proportion and is assumed as 50% (0.5) since there is no study found in Ethiopia.

Level of significance = 5% (α = 0.05), *Z*α/2 = 1.96
Margin of error = 5% (d = 0.05)
Non-response rate = 10%
Applying the formula, n = (*Z*α/2)2 * P (1−P)/d2

Then, n = $\frac{\left(1.96)2[0.5*(1-0.5)\right]}{(0.05)2}=384$ . By adding the 10% non-response rate, the sample size will be 423.

### Sampling procedure

The average number of people who use holy water at the Andassa Saint George Monastery was 20,280/year, equivalent to 1,690 people coming to the Andassa Saint George Monastery per month.

Systematic random sampling was used to select study subjects. The interval size (*k*) was calculated using the following formula: *k* = *N*/*n* = 1,690/423 = 3.995 ≈ 4, such that every four people were selected after the first subject selected by the lottery method.

### Data collection tools and procedure

Data were collected by six BSc nurses using electronic application for 1 month and supervised by two Msc students in ICCMH. Even though there is no separate room, the interview was conducted in a separate private place. A semi-structured sociodemographic interviewer-administered questionnaire was used to obtain data such as age, sex, ethnicity, marital status, educational attainment, employment status, income, and residence. The structured questionnaire of suicide behavior questionnaire revised (SBQ-R) was used to asses suicide.

**SBQ-R:** SBQ-R is a very useful tool to measure the different dimensions of suicidality and has four items:

Item 1 (4 points): evaluates lifetime suicidal ideation/suicidal plan/suicide attempt.
Item 2 (5 points): the frequency of suicidal ideation over the past 12 months.
Item 3 (3 points): the threat of suicide attempt.
Item 4 (6 points): the SBQ-R contains four items on suicidal behaviors, and the total score of the SBQ-R ranges from 3 to 18.

In a previous study, a sensitivity of 0.93 and a specificity of 0.95 indicate the risk of suicide in a general population (39). The SBQ-R is also approved as a useful scale for suicide risk assessment in clinical and non-clinical samples and is the most utilized tool (40).

**PHQ-9**: Depressive symptoms were assessed using the nine-item patient health questionnaire (PHQ-9), which was developed to assess probable depression in the primary care setting. The PHQ-9 could be used as a continuous scale, or the scores may be categorized into severity grades and individual scores at a cut point of 10 or more out of nine items in the patient health questionnaire. The PHQ-9 has been validated in the general hospital setting in Ethiopia, as well as in primary care settings in preparation for the current study (41).

**Generalized Anxiety Disorder-7 (GAD-7):** It is the most used tool for screening of anxiety by remembering the past 2 weeks. It also contains seven items with a four-Likert item. The tool is cross-culturally validated with the internal consistency of Cronbach’s α = 0.915. A score greater than or equal to 10 is considered as having moderate to severe anxiety disorder. Each item is scored from 0 (not at all) to 4 (nearly every day).

The scores of all items are added to obtain the total scores, with a range of 0–21. Those who scored more than 9 on the GAD-7 scale were diagnosed with anxiety (42, 43).

**The Somatic Symptom Scale-8 (SSS-8**): It is an abbreviated eight-item version of the Patient Health Questionnaire-15 (PHQ-15) questionnaire. The PHQ-15 scale, one of the most commonly used and most thoroughly validated self-report measures of somatic symptom burden, evaluates the presence and severity of common somatic complaints. The SSS-8 is a reliable and valid self-report measure of somatic symptom burden. It has an acceptable internal consistency (α = 0.806).

**Alcohol use disorder:** Alcohol consumption in the past 12 months was assessed using the Alcohol Use Disorder Identification Tool (AUDIT), a 10-item screening tool developed by the WHO. Each item was rated on a five-point scale, with the total score ranging from 0 to 40. A score of 20 or more on the AUDIT requires further diagnostic assessment for possible alcohol dependence. A total score of 1–7 denotes social drinking, and a total score of 8 or more indicates probable alcohol use disorder; a total score of 8–15 indicates hazardous alcohol use, a total score of 16–19 indicates harmful alcohol use, and a total score of 20 or more indicates probable alcohol dependence in the last 12 months (44). The AUDIT was found to have a high internal consistency in this study (Cronbach’s α = 0.84) (45).

**Problematic khat use:** PKUST-17 is a problematic khat use screening test with 17 items focusing on the frequency of khat use, the amount of time spent chewing khat, financial problems, and different withdrawal experiences. The internal consistency of PKUST-17 was excellent (Cronbach’s α = 0.93) (46).

**Smoking**: The Fagerstrom Test for Nicotine Dependence (FTND) was used to assess tobacco dependence. At a cutoff score ≥5, the FTND has good sensitivity and specificity (0.75 and 0.80, respectively). In a study conducted at the Jimma University Teaching Hospital, a total score of FTND ≥5 was considered to indicate tobacco dependence (47).

**Social support**: Social support was assessed by using the Oslo 3 Social Support Scale (OSSS-3). It has been used in different clinical and community-based studies of African countries including Ethiopia. It has a total sum score ranging from 3 to 14 points (48).

### Data entry, processing, and analysis

First, the collected data using the screening tools were cleaned and checked for completeness, coded, entered using epi data 5, and then exported into SPSS version 25 for analysis. Basic descriptive and summary statistics was computed by using means, standard deviation, percentages, proportions, and data frequencies. The findings were presented in the form of tables and charts. A binary logistic regression model was employed to determine statistical association between the independent and the dependent variables with the confidence interval set at 95%. All variables associated with the dependent variable with a *p*-value of less than 0.25 in the bivariate analyses of the binary logistic regression were entered into multivariate models of the logistic regression in order to identify the interaction between variables and to control for potential confounders. Variables with *p*-value < 0.05 in the multivariate analyses were considered as significant predictors of suicidal behavior.

## Results

### Sociodemographic characteristics of the study participants

A total of 423 respondents were included in the study, with a response rate of 100%. Regarding the age of the respondents, 118 (27.9%) were 18–24 years old, and 150 (35.5%) were 45 years and older. Among respondents, 224 (53.2%) were male, 422 (99.8%) were orthodox, 149 (35.5%) were not educated, and 79 (19.6%) were diploma holders and above. Of the participants, 187 (44.2%) were single and 142 (33.6%) were married; 281 (66.4%) lived with family; and 108 (25.5%) lived alone, as shown in **Table 1**.

**Table 1:** Frequency distribution of sociodemographic characteristics of holy water users at the Andassa Saint George Monastery, Northwest, Ethiopia, 2023 (*n* = 423).

Variables	Categories	Frequency	Percentage
Sex	Female	199	47%
	Male	224	53%
Age	18–24	118	27.9%
	25–34	60	14.2%
	35–45	95	22.5%
	45 and above	150	35.5%
Religion	Orthodox	422	99.8%
	Muslim	1	0.2%
Educational status	Not educated	149	35.2%
	Primary school	112	26.5%
	Secondary school	83	18.7%
	Diploma and above	79	19.6%
Income	Low	273	64.5%
	Medium	104	24.6%
	High	46	10.9%
Marital status	Married	142	33.6%
	Single	187	44.2%
	Divorced/widowed	94	22.2%
Living circumstances	With family	281	66.4%
	With other social	34	8%
	Alone	108	25.5%

### Clinical related factors of the study participants

Out of the total participants, 55 (13%) had a family history of mental illness, and 14 (3.3%) had a family history of suicidal behavior; the result also showed that 28 (6.6%) had hypertension, 7 (1.7%) had TB, 33 (7.8%) had DM, 17 (4%) had HIV/AIDS, 23 (5.7%) had epilepsy, 6 (1.4%) had cardiac disease, and 5 (1.2%) had cancer. Regarding mental illness, depression accounted a prevalence of 98 (23.2%), anxiety 60 (14.2%) and Somatic symptom accounted 37(8.7%) high and 59(13.9%) very highly (**Table 2**).

**Table 2:** Frequency distribution of mental illness and suicidal behavior in the family, the chronic medical illness, and mental illness among holy water users at the Andassa Saint George Monastery, Northwest Ethiopia, 2023 (*n* = 423).

Variables	Categories	Frequency	Percentage
Family history of mental illness	Yes	55	13%
	No	368	87%
Family history of suicide	Yes	14	3.3%
	No	409	96.7%
Hypertension	Yes	28	6.6%
	No	398	93.4%
DM	Yes	33	7.8%
	No	390	92.2%
HIV/AIDS	Yes	17	4%
	No	406	96%
Epilepsy	Yes	23	5.4%
	No	400	93.3%
	No	363	85.8%
Cardiac illness	Yes	6	1.4%
	No	417	98.6%
Depression	Yes	98	23.2%
	No	325	76.8%
Anxiety	Yes	60	14.2%
	No	363	85.8%
Somatic symptom	No	199	47%
	Mild	84	19.9%
	Moderate	44	10.4%
	Severe	37	8.7%
	Highly severe	59	13.9%

### Social support and substance-related factors

This study revealed that the majority of the participants received moderate social support, 171 (40.4%); poor social support, 98 (23.2%); and strong social support, 154 (36.4%).

Of the participants related to substance use, 153 (36.2%) had probable alcohol use disorder; 39 (9.2%), hazardous alcohol use disorder; and 17 (4%), harmful alcohol use disorder. Regarding nicotine-related disorders, there are 3 (0.7%) mild and 1 (0.2%) highly severe users. Of the total participants 10(2.36%) had history of chewing khat last three months (**Table 3**).

**Table 3:** Frequency distribution of the substance history among holy water users at the Andassa Saint George Monastery, Northwest Ethiopia, 2023 (*n* = 423).

Variables	Categories	Frequency	Percentage
Alcohol	Social drinker	214	50.6%
	Probable alcohol use disorder	153	36.2%
	Hazardous alcohol use	39	9.2%
	Harmful alcohol use	17	4.%
	Probable alcohol dependence	1	0.2%
Nicotine	Never	419	99.1%
	Mild	3	0.7%
	Highly severe	1	0.2%
Khat	Yes	10	2.3%
	No	413	97.7%

### The prevalence of suicidal behavior among respondents

**Figure 1:**
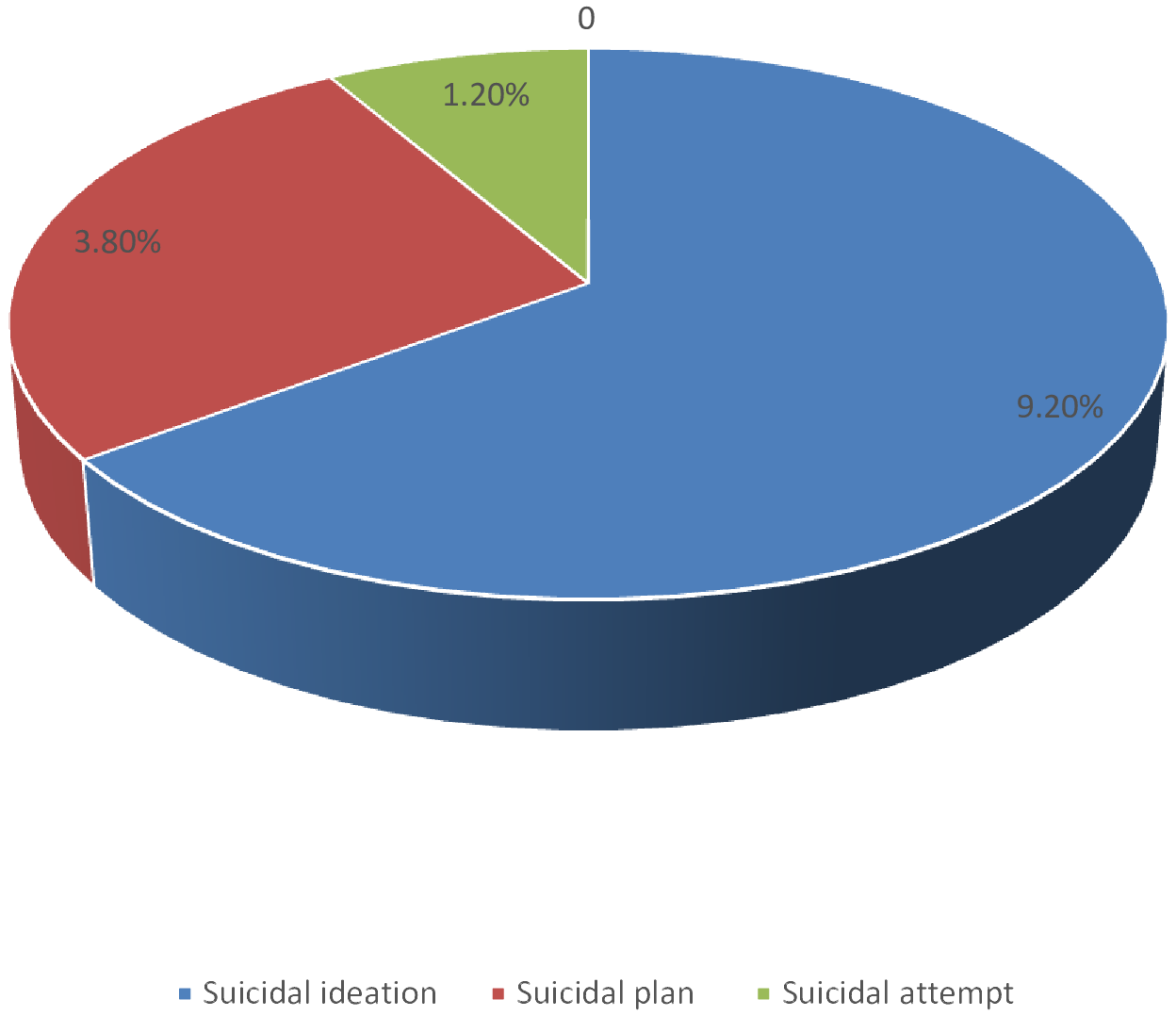
Frequency distribution of the suicidal behavior among holy water users at Andassa Saint George Monastery, northwest Ethiopia, 2023 (n=423).

According to this study, the lifetime prevalence of suicidal behavior among holy water users was 9.7% (95% CI: 7.1–12.3), and it was highly prevalent among women (6.6%). The lifetime prevalence of suicidal ideation, plan, and attempt was 39 (9.2%), 16 (3.8%), and 5 (1.2%), respectively, as shown in **Figure 1**.

### Suicidal behavior and associated factors

To determine the association of independent variables with suicidal behavior, bivariate and multivariate binary logistic regression analyses were carried out. On the bivariate analysis of suicidal behavior in relation to each variable, being female, age 18–24 years, living alone, being single, poor social support, having chronic medical illness, and having depression and anxiety were significant at a *p*-value less than 0.25. These factors were entered into multivariable binary logistic regression for further analysis in order to control confounding effects.

In multivariate analysis, being female, living alone, having chronic medical illness, and having depression were significantly associated with suicidal behavior at *p*-values equal to or less than 0.05.

Being female; living alone; having chronic medical illness like cancer, epilepsy, and DM; and having depression were factors associated with suicidal behavior.

Being female was 2.63 times more likely to have suicidal behavior than being male (AOR = 2.63, 95% CI: 1.20–5.74). Individuals who live alone were 2.54 times more likely to have suicidal behavior than individuals who live with their families (AOR = 2.54, 95% CI: 1.06–597). Participants who have epilepsy were 3.82 times more likely to have suicidal behavior than people without epilepsy (AOR = 3.816, 95% CI: 1.28–11.40). Individuals with DM were 3.371 times more likely to have suicidal behavior than people without DM (AOR = 3.371, 95% CI: 1.229–9.25), and people with depression were 3.03 times more likely to have suicidal behavior than people without depression (AOR = 3.03, 95% CI: 1.32–6.99) (**Table 4**).

**Table 4:** The bivariable and multivariable logistic regression analysis results of suicidal behavior and associated factors among holy water users at the Andassa Saint George Monastery, Northwest Ethiopia, 2023 (*n* = 423).

Variables	Categories	Suicidal behavior		COR (95% CI)	AOR (95% CI)
		Yes	No		
Educational level	Diploma and above	5	74	Ref	
	Secondary school	12	71	2.51 (0.84–7.46)	
	Primary school	10	102	1.45 (0.48–4.42)	
	Not educated	14	135	1.54 (0.53–4.43)	
Sex	Male	13	211	Ref	Ref
	Female	28	171	2.66 (1.34–5.29)	2.632 (1.21–5.75)**
Age	45 years and above	7	143	Ref	Ref
	35–44 years	10	85	2.40 (0.88–6.55)	
	25–34 years	7	53	2.69 (0.90–8.06)	
	18–24 years	17	101	3.44 (1.38–8.59)	1.85 (0.38–8.94)
Marital status	Married	7	135	Ref	Ref
	Divorced/widowed	6	88	1.32 (0.43–4.04)	
	Single	28	159	3.34 (1.44–8.02)	
Living circumstances	With family	20	261	Ref	Ref
	With other social	4	30	1.74 (0.56–5.43)	
	Living alone	17	91	2.44 (1.22–4.85)	2.52 (1.06–5.97)*
Income	High	8	38	Ref	Ref
	Medium	6	98	0.34 (0.12–1.01)	
	Poor	26	247	0.50 (0.21–1.18)	
History of mental illness in the family	Yes	9	46	Ref	Ref
	No	32	336	2.054 (0.922–4.577)	
History of suicide in the family	Yes	3	11	Ref	Ref
	No	38	371	2.66 (0.71–9.96)	
Hypertension	Yes	3	25	Ref	Ref
	No	38	357	1.13 (0.32–3.91)	
HIV/ADS	Yes	4	13	Ref	Ref
	No	37	369	3.07 (0.95–9.89)	
Epilepsy	Yes	7	16	Ref	Ref
	No	34	366	4.71 (1.81–12.24)	3.82 (1.28–11.40)*
DM	Yes	9	24	Ref	Ref
	No	32	358	4.19 (1.79–9.78)	3.37 (1.23–9.25)*
Depression	Yes	22	76	Ref	Ref
	No	19	305	4.65 (2.39–9.02)	3.03 (1.32–6.99)**
Anxiety	Yes	13	47	Ref	Ref
	No	28	335	3.31 (1.60–6.83)	2.03 (0.79–5.17)
	No	14	185	Ref	Ref
	Mild	12	72	2.20 (0.97–4.98)	
	Moderate	6	38	2.08 (0.75–5.77)	
	Severe	2	35	0.75 (0.16–3.47)	
	Highly severe	7	52	1.78 (0.68–4.64)	
Social support	Strong social support	11	143	Ref	Ref
	Moderate social support	15	156	1.25 (0.55–2.81)	
	Poor social support	15	83	2.35 (1.03–5.35)	1.78 (0.69–4.54)
Alcohol use	Never	23	191	Ref	Ref
	Mild	14	139	0.79 (0.38–1.62)	
	Moderate	2	37	0.44 (0.10–1.98)	
	Severe	2	15	1.11 (0.24–5.15)	

Ref, Reference; *p< 0.05, **p< 0.01.

## Discussion

This institution-based cross-sectional study design aimed to assess the prevalence of suicidal behavior and its associated factors among holy water users at the Andassa George’s monastery in the Amhara region. This study found that lifetime prevalence of suicidal behavior was 9.7% with 95% CI (7.1–12.4). The prevalence of suicidal ideation, plan, and attempt was 9.2%, 3.8%, and 1.2%, respectively.

The results of the study were consistent with a study done in Gedeo Zone, southern Ethiopia, among pregnant mothers (9.3%) (49), a study conducted among patients attending an emergency department (8.8%) (50), and a study conducted in Brazil (9.10%) (50).

This result was higher than that of a study conducted among patients and residents in Northwest Ethiopia (5.6%) (50) and a study conducted in Butajira, Ethiopia with a lifetime suicide attempt of 3.2% (50). The probable variation might be attributed to the different study population, which, in the case of this study, was conducted among holy water users, who may suffer from physical and mental illnesses and who are separated from their family and thus may experience adjustment problems.

This result was lower than that of the study conducted among prisoners in Dessie Town (25.3%; 95% CI: 20.5–30.6) (51), the study conducted among Mettu University students (28.9%; 95% CI: 25.0–32.5) (52), and the study conducted in Southern Ethiopia among street-living homeless young people (38.2%; 95% CI: 34.8–41.5) (50). The possible reasons might be due to the difference in study population: the study population in Dessie consisted of prisoners who were high risk of having antisocial personality and some sort of mental illness; university students in Mettu (both graduate and freshman students) experience significant amounts of stress and separation anxiety, and their suicidal behavior is strongly characterized by depression, hopelessness, and desperation (39); and homeless young people in Southern Ethiopia suffer from a high level of severe mental illness and poor social support, and in the general, higher levels of religious involvement are positively associated with indicators of psychological wellbeing (life satisfaction, happiness, positive affect, and higher morale) and with less depression (53).

As regards associated factors, female respondents were 2.63 times more likely than male respondents to engage in suicidal behavior. The reason for this could be that women are more vulnerable to psychosocial stressors, have poor coping mechanisms, and are more likely to have depression compared to men; hence, women have a higher rate of suicidal behavior than men (54). The other possible reason could be related to biological differences, sociocultural influences on women’s ability to articulate their difficulties in comparison to men, and suppressed emotional expression, which can contribute to high suicidal tendencies (55). Among associated factors of suicidal behavior, living alone makes respondents 2.53 times more likely to have suicidal behavior than respondents who live with family. This might be due to the fact that those who lived alone had no nearby family with whom they could communicate and share their problems, which could lead to increased hopelessness and suicidal behavior among people who live alone (56), which is supported by a study conducted in the South Gondar zone, Northwest Ethiopia (57) **and residents** in Northwest Ethiopia (58).

Suicidal thoughts are significantly associated with chronic medical conditions (59). In the present study, participants who reported having epilepsy were 3.82 times more likely to engage in suicidal behavior than those who had no epilepsy. Epilepsy-related medical and mental comorbidities, the combined impact of illness and medication side effects, stigma associated with epilepsy or perceived stigma, frequent seizures linked to depression, and social disadvantage and stigma in people with epilepsy are all potential contributors to suicidal behavior (55). This study was in agreement with studies conducted in Addis Ababa, Ethiopia (60).

Another chronic medical illness associated with suicidal behavior in this study was DM. Individuals with DM were 3.37 times more likely to have suicidal behavior than people without DM. The following might be the possible reasons for this: pain secondary to medical illness, lifetime use of some medications used for chronic medical illness, disruption of relationships, functional impairment due to illness that may predispose the individual to socioeconomic difficulties, and depression or stress that increases the risk of suicidal behavior. Another possible reason is that glycemic control might be a potential clinical mediator of the relationship between suicidal behavior and diabetes complications (48). It is supported by studies conducted in Felege Hiwot Referral Hospital, Bahirdar, Ethiopia (39).

In this study, having depression was found to be highly associated with suicidal behavior in the current study. Participants with depression were 3.03 times more likely to engage in suicidal behavior than those without depression. Depression is a prevalent psychiatric disorder characterized by a loss of interest in oneself, others, and the future, all of which leads to self-harming thoughts and actions.

This could be due to lower amounts of the neurotransmitter serotonin in the brain in depressed individuals, which can lead to emotions of hopelessness, worthlessness, and guilt, which can contribute to suicidal behavior (55).

This is consistent with prior studies conducted among rural community members of the Gedeo zone in southern Ethiopia (32), Mettu University, Southwest Ethiopia (61), and patients and residents of Northwest Ethiopia (62).

## Conclusions

In this study, almost 1 in 10 had engaged in suicidal behavior in their lifetime. Several risk factors for suicidal behavior were identified, including being female, living alone, and having chronic medical and mental illness. As a result, early screening, detection, and management of suicidal behavior and treating identified associated factors will minimize the burden of suicide among holy water users. Therefore, health professionals should create awareness about suicide, its signs and symptoms, and factors associated with suicidal behavior for religious leaders/fathers. Mental health professionals should work in collaboration with religious leaders/fathers on screening and managing suicidal behavior. It is better to include and implement assessment of suicidal risk factors as a primary focus on holy water users and community training regarding suicidal behavior. Our findings help in terms of being a primary supporting evidence for health policymakers to recommend integration of mental health services within traditional treatments and assisting in planning suicide prevention in holy water settings, by integrating modern mental health services with traditional mental health services such as at monasteries and/or starting community educational programs.

### Limitation of the study

Since face-to-face interview was the primary data collection method and there was no room to interview patients, there may have been privacy issues.

There could also have been social desirability bias to disclose some information that is perceived as a socially/religiously sensitive issue. In this study, it is difficult to create a causal association between the outcome variable and predictors because we used a cross-sectional study design.

## Data availability statement

The original contributions presented in the study are included in the article/Supplementary Material. Further inquiries can be directed to the corresponding author.

## Ethics statement

The studies involving humans were approved by Bahir Dar university institutional review board (on April 20/2023 with the protocol number of 777/2023). The studies were conducted in accordance with the local legislation and institutional requirements. The participants provided their written informed consent to participate in this study. All information generated from the study was treated with confidentiality and was reported as a group data summary without disclosing any potentiality of identifying information for any research participant and referral was given for positive cases for suicidal behavior.

## Author contributions

GK: Conceptualization, Investigation, Methodology, Software, Visualization, Writing – original draft, Writing – review & editing. FA: Conceptualization, Investigation, Methodology, Writing – original draft, Writing – review & editing. TB: Conceptualization, Investigation, Methodology, Writing – original draft, Writing – review & editing. HB: Conceptualization, Investigation, Methodology, Software, Writing – original draft, Writing – review & editing. BM: Conceptualization, Investigation, Methodology, Software, Supervision, Writing – original draft, Writing – review & editing.
